# The Resolution of Intestinal Inflammation: The Peace-Keeper’s Perspective

**DOI:** 10.3390/cells8040344

**Published:** 2019-04-11

**Authors:** Sara Onali, Agnese Favale, Massimo C Fantini

**Affiliations:** 1 Department of Biomedicine and Prevention, University of Rome “Tor Vergata”, Via Montpellier 1, 00133 Rome, Italy; sara.onali@uniroma2.it; 2 Department of Systems Medicine, University of Rome “Tor Vergata”, Via Montpellier 1, 00133 Rome, Italy; agnesefavale@icloud.com

**Keywords:** colitis, resolution, immunosuppression, counter-regulatory mechanisms

## Abstract

The uncontrolled activation of the immune system toward antigens contained in the gut lumen in genetically predisposed subjects is believed to be the leading cause of inflammatory bowel disease (IBD). Two not mutually exclusive hypotheses can explain the pathogenic process leading to IBD. The first and mostly explored hypothesis states that the loss of tolerance toward gut microbiota antigens generates an aberrant inflammatory response that is perpetuated by continuous and unavoidable exposure to the triggering antigens. However, the discovery that the resolution of inflammation is not the mere consequence of clearing inflammatory triggers and diluting pro-inflammatory factors, but rather an active process in which molecular and cellular elements are involved, implies that a defect in the pro-resolving mechanisms might cause chronic inflammation in different immune-mediated diseases, including IBD. Here we review data on pro-resolving and counter-regulatory mechanisms involved in the resolution of inflammation, aiming to identify their possible involvement in the pathogenesis of IBD.

## 1. Introduction

In normal conditions, the immune homeostasis in the gut is tightly regulated by the balance between pro- and anti-inflammatory mechanisms to avoid undesired immune response against harmless antigens produced by the intestinal microbiota or introduced by diet. However, the mucosal immune system is in charge of identifying pathogens and reacting against them with a protective, self-limiting, immune response. Crohn’s disease and ulcerative colitis, the two major forms of inflammatory bowel disease (IBD), are believed to be the consequence of an unnecessary immune reaction toward microbiota-derived antigens normally contained in the gut of genetically susceptible subjects [1]. Two not mutually exclusive hypotheses can explain the chronic inflammation occurring in IBD patients. According to the first and most accredited hypothesis, loss of tolerance toward microbiota-derived antigens triggers mucosal inflammation, which is in turn chronically maintained by continuous and unavoidable exposure to the initiating antigens. However, according to a second hypothesis, an initial inflammatory flare induced by luminal antigens fails to resolve because of defects in the counter-regulatory and pro-resolving systems.

Inflammation was clinically described in the first century by Hippocrates who defined *rubor*, *calor*, *tumor*, and *dolor* the main features of this process. We now know that vasodilation, associated with increased vascular permeability and accumulation of inflammatory cells in the stroma, characterizes the earliest phases of inflammation. We also know that the progressive activation of innate and adaptive immunity is associated with the expression of a plethora of pro-inflammatory mediators responsible for the activation of several mechanisms able to clear the *noxa* that triggered the immune response. Each of these phases is constantly kept in check by counter-regulatory mechanisms, in order not only to avoid an over-response leading to collateral tissue damage but also to actively promote the resolution of the inflammatory process. Indeed, accumulating evidence indicates that the resolution of the inflammatory process is not the mere consequence of clearing inflammatory triggers and diluting pro-inflammatory factors but an active process that involves molecular and cellular mechanisms initiated at the onset of inflammation [2]. The concept that the resolution of inflammation is an active mechanism highlights the differences to immunosuppression, with potentially relevant consequences for therapy. Indeed, while resolution is considered a physiological part of the inflammatory process, immunosuppression results from the blockage of a specific step in the pro-inflammatory cascade. From this perspective, immunosuppression limits inflammation and inflammation-induced collaterals by breaking a single link in the immune response chain of events (e.g., Tumor necrosis Factor (TNF)-alpha neutralization by anti-TNF monoclonal antibodies), neglecting to restore other systems whose persistent alteration might continue to fuel the inflammatory process. Accordingly, inflammatory bowel diseases are affected by a high rate of relapse after biological and immunosuppressive therapy suspension [3]. In contrast, the re-activation of pro-resolving mechanisms might induce more profound and long-lasting anti-inflammatory effects by regulating a wider range of immune counter-regulatory mechanisms. In this context, the re-evaluation of the already characterized counter-regulatory mechanisms from a pro-resolving perspective might be useful for identifying new pathogenic mechanisms and to envision new therapeutic strategies. Here, we review data on immune-suppressive and regulatory mechanisms involved in the gut immune homeostasis, focusing on their involvement in pro-resolving mechanisms and the resolution of intestinal inflammation.

## 2. Specialized Pro-Resolving Molecules (SPMs) and Resolution of Inflammation

**Figure 1:**
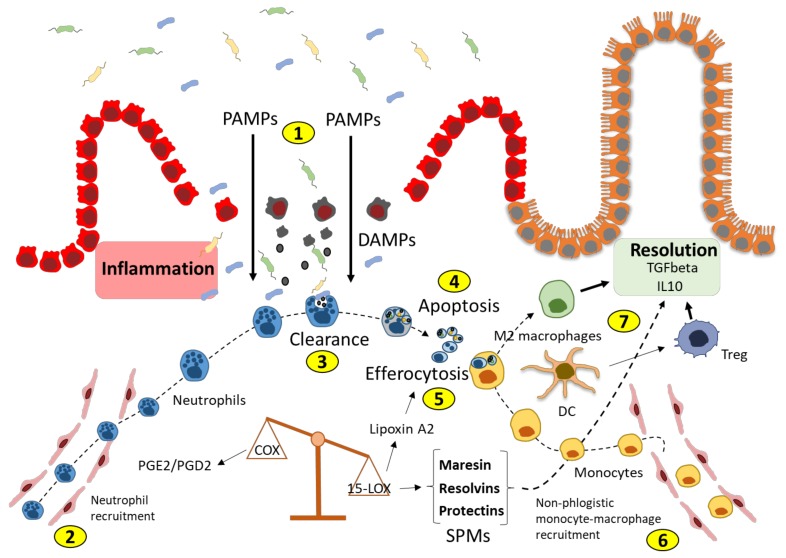
The pro-resolving roadmap. In the presence of pathogen-associated molecular patterns (PAMPs) and/or damage-associated molecular patterns (DAMPs), (1) the cyclooxygenase (COX)-mediated production of prostaglandin (PG) E2 and D2 recruits neutrophils (2) responsible for the clearance of pathogens and cellular debris (3). In a pro-resolving setting, after engulfment, neutrophils undergo apoptosis (4) which promotes efferocytosis (5) by non-phlogistic monocyte–macrophages recruited by 15-lipoxigenase (15-LOX)-generated specialized pro-resolving mediators (SPMs) (6). Efferocytosis in macrophages and dendritic cells (DCs) induces a pro-resolving response characterized by the differentiation of anti-inflammatory M2 macrophages and suppressive regulatory T cells (Tregs) (7).

The beginning of the inflammatory process is characterized by a coordinated series of events. Depending on the nature of the initiating trigger (e.g., intracellular or extracellular pathogens, parasites, ischemia/reperfusion injury, trauma), resident cells, including epithelial cells, dendritic cells, and innate lymphoid cells, initiate neutrophil recruitment through the post-capillary venules to phagocytize invading microbes and cellular debris [4]. When excessive, the accumulation of neutrophils leads to the undesired release of antimicrobial armamentarium and lytic enzymes via frustrated phagocytosis [5] or necrosis, thus inducing tissue collateral damage and the release of damage-associated molecules (DAMPs), that, in association with pathogen-associated molecules (PAMPs) derived from invading microbes, lead to the activation of the inflammasome and progression to chronic inflammation [6]. In contrast, neutrophil apoptosis was shown to inhibit Toll-like receptor (TLR) signaling and inflammasome activation [7]. A key event in the resolution process is represented by the non-phlogistic monocyte–macrophage recruitment at the site of inflammation (Figure 1). After the initial inflammatory influx of neutrophils, macrophages scavenge apoptotic neutrophils and tissue debris, a process called efferocytosis (from Latin *efferre*, meaning “to bury”), thus progressively reducing pro-inflammatory stimuli. A switch occurring in the arachidonic acid metabolism mediates the passage from neutrophil-to-non-phlogistic monocyte–macrophage recruitment. Indeed, during the early phases of inflammation, cycloxigenase-derived prostaglandin E2 (PGE2) and Prostaglandin D2 (PGD2), which are involved in the process of neutrophil recruitment, induce the expression of 15-lipoxigenase (15-LOX) switching to lipoxin production. Lipoxins limit the further recruitment of neutrophils at the site of inflammation [8]. Lipoxin A2 also stimulates macrophage efferocytosis, thus marking the passage to the resolution phase [9]. In addition to lipoxins, many derivatives of eicosapentaenoic acid (EPA), docosapentaenoic acid (n-3DPA), and docosahexanoic acid (DHA) metabolism have been shown to play multiple non-redundant roles in the resolution of inflammation. These metabolites have been grouped into three distinct families of specialized pro-resolving mediators (SPMs): resolvins (short for “resolution phase interaction products”), protectins, and maresins (short for “macrophage mediators in resolving inflammation”). Four human SPM receptors have been identified: ALX/FPR2, ERV1, DRV1, and DRV2. Although initially recognized as receptors for Lipoxin A4, Resolvin E1, Resolvin D1, and Resolvin D2, respectively, each receptor has been shown to interact with additional SPMs [10]. The downstream intracellular signaling evoked by SPMs includes the activation of Erk1/2, AKT, JNK, and Caspase 3-mediated apoptosis [11,12].

SPMs are differentially expressed during the inflammatory process, and different functions have been identified for several of them in models of infection and sterile inflammation. In a murine model of *Escherichia coli*-induced peritonitis, resolvin (Rv) D1 and RvD5 reduced both blood and exudate bacterial titers by increasing neutrophil and macrophage phagocytosis [13]. Moreover, RvD1 in addition to ciprofloxacin was more effective than ciprofloxacin alone in reducing blood bacterial titers. RvD1 was also associated with reduced levels of the pro-inflammatory cytokines IL-1beta and IL-6 and with increased IL-10 and IFN-gamma expression. In human macrophages, RvD1, RvD5, and protectin PD1 increased phagocytic activity and reactive oxygen species (ROS) while reducing Nuclear factor kB (NFkB) activation and TNF-alpha expression [13]. Similarly, RvD1 induced efferocytosis, accelerating neutrophil and bacterial clearance in *E. coli* and *Pseudomonas aeruginosa*-mediated pneumonia models [14]. RvD2 was found to limit neutrophil infiltration and to enhance bacteria clearance by phagocytes in models of *E. coli* and *Staphylococcus aureus* infection. In sterile models where inflammation was induced by ischemia–reperfusion injury, RvD2 decreased neutrophil infiltration of the lungs and induced the production of other SPMs in a RvD2-receptor-dependent manner [15]. Maresin (MaR) 1 followed by RvD1 and RvD3 are produced in the lungs after exposure to inhaled gastric acid. Intravenous administration of these SPMs limits the severity of acute inflammation and promotes a more rapid resolution [16,17,18].

In addition to myeloid cells, SPMs have been shown to act on cells of lymphoid origin. In patients affected by asthma, natural killer (NK) cells and type 2 innate lymphoid cells (iLC2) are involved in the containment of eosinophil and antigen independent IL-13 expression, respectively. Lipoxin A4 increased NK-mediated apoptosis of eosinophils and decreased IL-13 expression by iLC2s in an ALX/FPR2-dependent manner [19]. In a murine model of asthma, MaR1 reduced both IL-5 and IL-13 expression in iLC2 and this was associated with an increased TGF-beta-dependent generation of regulatory T cells (Tregs) and Tregs-mediated suppression of iLC2 [20]. RvD1, RvD2, and MaR1 were shown to reduce cytokine expression by CD8+ and CD4+ Th1 and Th17 cells. Reduced expression of IFN-gamma and IL-17A, together with the Th1 and Th17 signature transcription factor Tbet and RORc expression, was associated with an increased expression of Treg-associated markers (i.e., FoxP3. Granzyme, CTLA-4) and IL-10 expression [21]. RvD1 was shown to induce Tregs and to resolve inflammation in a model of experimental autoimmune neuritis [22]. Finally, SPMs have an adjuvant effect increasing IgM and IgG production in CD27+CD38+ antibody-secreting B cells [23]. In patients affected by chronic heart failure (CHF), a condition characterized by chronic inflammation, T cells showed reduced production of RvD1. Moreover, CD8+ and CD4+ T cells isolated from peripheral blood mononuclear cells of CHF patients failed to downregulate the expression of TNF-alpha, IFN-gamma, IL-17, and IL-2 in response to RvD1 and RvD2 stimulation due to low expression of the resolvin receptor GPR32 [24]. 

## 3. SPMs in Inflammatory Bowel Disease

Data from animal models of colitis support the role of SPMs in the resolution of intestinal inflammation. Initial studies demonstrated that transgenic mice characterized by high endogenous synthesis of n-3 PUFA and enriched in the SPM precursors EPA and DHA were protected from colitis induced by oral administration of dextran sulphate sodium (DSS). Since then, SPMs have been shown to prevent experimental colitis in different models (Table 1). However, the demonstration that SPMs can induce the resolution of the inflammatory process came from the observation that MaR1 improved established chronic colitis induced by multiple DSS administrations [25]. Moreover, the role of SPMs in mucosal healing was analyzed by lipidomic analysis in mice developing DSS-mediated colitis. In this model, mucosal healing was promoted by the specific down-regulation of pro-inflammatory lipidic mediators, associated with increased production of RvD1 and a decrease of its precursor, DHA, as well as the resolvin E precursor 18-hydroxy-EPA [26]. These data suggest that in analogy to what has been observed in other organs, the switch from pro-inflammatory to pro-resolving lipidic mediators marks the initiation of the resolution phase in the intestinal mucosa. Despite the evidence that SPMs might be involved in the resolution of intestinal inflammation, the data failed to demonstrate a defect of SPM production in IBD. Indeed, RvD5 and PD1 were upregulated in biopsies from IBD patients, and in addition to their protective role in the DSS model of colitis, they were shown to reduce cell adhesion in TNF-alpha-treated human endothelial cells in vitro [27]. However, whether a defect in SPM signaling exists in IBD, as shown in CHF, remains to be explored.

## 4. Neutrophil Apoptosis, Efferocytosis, and Macrophage Plasticity as Pro-Resolving Mediators

As mentioned above, apoptosis of neutrophils which have accumulated at the site of inflammation in order to eliminate pathogens is crucial for the resolution of the inflammatory process. The expression of “eat-me” signals by apoptotic cells induces efferocytosis by resident and monocyte-derived professional phagocytes [28], and the engulfment of apoptotic neutrophils results in engulfing cells re-programming in order to promote the resolution of inflammation. If neutrophils fail to undergo apoptosis or if they are not cleared properly, inflammation persists and tissue collateral damage occurs.

Different “eat-me” signals have been thus far identified. The first and most investigated one is phosphatidylserine (PS). PS is a phospholipid localized, in living cells, in the inner layer of the cellular membrane leaflet. In apoptotic cells, PS is exposed externally where it interacts with different receptors grouped in four major families (i.e., BAI, TIM, Stablin, and CD300, reviewed elsewhere [29]) to mediate efferocytosis. In a model of peritonitis, classically activated monocyte-derived M1 macrophages characterized by the expression of inducible Nitric oxide synthase (iNOS) and pro-inflammatory cytokines and chemokines in response to IFN-gamma and TLR ligands, undergo reprogramming, leading first to the acquisition of an M2-like phenotype characterized by high efferocytosis and then to the concomitant expression of pro-inflammatory cytokines and IL-10 [30]. When the engulfing activity is at maximum, the exhausted phagocytes are further reprogrammed, leading to the generation of deactivated pro-resolving macrophages which express TGF-beta and IL-10, downregulate MHC class II, and exert immunosuppressive functions [30]. In addition, efferocytosis induces the 15-lipoxigenase-dependent synthesis of pro-resolving lipids in macrophages, thus enhancing their anti-inflammatory function. Also, DCs have the capacity to engulf apoptotic neutrophils. DC-mediated efferocytosis results in the downregulation of CD11b, MHC, and CD86, thus reducing the DC antigen-presenting capacity. At the same time, the increased CCR7 expression addresses DC to lymphoid organs where they down-regulate the adaptive immune response [31]. The uptake of apoptotic cells induces the expression of TGF-beta, IL-10, and retinoic acid (RA) which promote the development of Tregs [32]. Induction of Tregs is also promoted by indoleamine 2,3 dioxygenase-1 (IDO-1), whose activity is induced by TGF-beta and apoptotic cell phagocytosis [33,34]. IDO-1 was shown to induce tolerance by inducing Treg differentiation, and studies have demonstrated that DC-mediated phagocytosis of apoptotic cells promotes the differentiation of naïve T cells into Tregs [35].

In normal conditions, the gut harbors the largest population of macrophages, and they are constantly replenished by the recruitment of Ly6C^high^ monocyte precursors from peripheral blood into the lamina propria in a CCR2-dependent manner [36,37]. This cell population, under the influence of the local environment, acquires an M2 phenotype that is pivotal in the maintenance of gut immune homeostasis through scavenger and bactericidal activities and in the production of the anti-inflammatory cytokine IL-10 [38]. During intestinal inflammation, as in the case of IBD, the monocyte recruitment is increased, but they fail to upregulate IL-10 and to become refractory to pro-inflammatory stimuli [36]. In this condition, monocytes acquire an M1 phenotype, becoming the main producers of pro-inflammatory mediators such as IL-1beta, IL-6, TNF-alpha, IL-23, NO, and ROS [39,40]. The reason why these cells fail to carry out efferocytosis and to reprogram into pro-resolving macrophages after apoptotic cell engulfment is unclear. However, in IBD, defective neutrophil migration at the site of inflammation has been observed. Patients affected by Crohn’s disease were found to have reduced accumulation of neutrophils in the colonic mucosa at sites where the mucosal barrier was mechanically broken by multiple biopsies [41]. A similar migration defect was observed in the skin of the same patients after subcutaneous injection of heat-killed *E. coli* as compared to healthy volunteers [41]. It is tempting to speculate that the defective migration of neutrophils in IBD patients might cause an inadequate accumulation of efferocytosis-inducing apoptotic bodies, thus leading to insufficient pro-inflammatory phagocyte reprogramming and perpetuation of the inflammatory process.

In addition to defects in neutrophil recruitment, other evidence suggests the involvement of defective efferocytosis in IBD pathogenesis. Experimental data indicate that efferocytosis is connected to a non-canonical form of autophagy, called LC3-associated phagocytosis (LAP), that involves LC3-lipidation of the phagosome and the activation of a series of factors (e.g., ATG5, ATG7, ATG12, ATG16L) which are shared with canonical autophagy [42]. The recognition of PAMPs by TLRs, the interaction between immunoglobulin receptors and the opsonized particles, and TIM4 binding to PS exposed on the surface of apoptotic bodies have been shown to induce LAP [43,44,45].

Allele variants of several genes involved in autophagy (e.g., ATG16L1, IRGM) and unfolded protein response (UPR) (e.g., XBP1) have been associated with an increased risk of developing IBD [46]. Most of the effects of these genetic variants have been linked to the metabolism of highly secreting intestinal epithelial cells in the gut, such as Paneth’s cells and goblet cells [47]. In these cells, the high production of secreted peptides induces, in genetically susceptible individuals, the accumulation of unfolded or misfolded proteins, endoplasmic reticulum (ER) stress, and, ultimately, apoptosis. Loss of Paneth’s cells and mucus-producing cells causes an irreversible alteration of the mucosal barrier, which is believed to sustain chronic inflammation. Apart from the role played by secreting cells, autophagy has been also linked to the clearance of intracellular pathogens by PRR. Indeed, NOD2 allele variants, associated with a high risk of developing CD, have been shown to promote the assembly of ATG16L1 and of other autophagy components at the intracellular site of pathogen entry [48]. IRGM have been shown to promote interaction between NOD2 and ATG16L1 and to induce xenophagy, a variant of the autophagy process used by cells to counteract intracellular pathogens [49].

Although efferocytosis has not been analyzed in IBD, its close relationship with autophagy, established by the co-sharing of several molecular factors involved in both processes, suggests that the same IBD-associated allele variants affecting autophagy might also induce a defect in efferocytosis and in the induction of pro-resolving macrophages in these patients.

In addition to PS, other receptors expressed on the surface of apoptotic neutrophils have been shown to play a role in stimulating efferocytosis, including the cytokine scavenger receptors D6 and CCR5 [50,51]. In the resolution phase of experimental peritonitis, D6-deficient mice showed an increased accumulation of apoptotic neutrophils in macrophages, which was associated with increased expression of TNF-alpha and IL-1beta. In this model, the absence of D6 did not impair the chemokine-scavenging activity, while a defect in reprogramming macrophages due to the altered interaction with apoptotic neutrophils was reported [51]. In another report, the interaction of D6 expressed by apoptotic neutrophils with CCR5 expressed on the macrophage cell membrane was dependent on the combined interaction with CCL5 and promoted macrophage reprogramming [52]. D6 expression increases during intestinal inflammation, and D6-deficient mice were protected from DSS-induced colitis. However, lamina propria mononuclear cells (LPMC) isolated from D6-deficient mice were characterized by persistent high expression of IFN-gamma, TNF-alpha, and IL-17A during the recovery phase according to the persistence of an underlying unresolved inflammatory process [53]. 

Annexin A1 is another pro-resolving mediator expressed on the surface of apoptotic neutrophils [54] but is also released as a component of extracellular microparticles and exosomes by both neutrophils and intestinal epithelial cells after injury [55,56]. The pro-resolving effect of Annexin A1 is mediated by interaction with ALX/FPR2 on responsive cells inducing neutrophil apoptosis, enhancing efferocytosis, macrophage reprogramming, and promoting proliferation of epithelial cells [56,57]. Annexin A1 has been shown to be released in severely inflamed areas of the colon of UC patients but not in mild UC or controls [58,59], and it is believed to play a role in the induction and maintenance of remission in these patients. In CD, elevation of Annexin A1 plasma levels was associated with response to therapy with the monoclonal anti-TNF antibody Infliximab [60]. According to this pro-resolving role in intestinal inflammation, Annexin A1-containing exosomes and microparticles have been shown to accelerate the process of mucosal healing by restoring the integrity of the intestinal epithelial barrier in in vitro and in vivo DSS models of colitis [56].

## 5. T Cells and Innate Lymphoid Cells as Pro-Resolving Mediators

Billions of microbial species and their products are constantly “sensed” by the immune system in the gut and the capacity to discriminate threads from harmless antigens determines the type of immune response evoked. Indeed, in the presence of pathogens, the adaptive immunity responds by inducing the differentiation of specialized classes of helper T cells. Th1 cells are generated to fight against intracellular pathogens, Th17 cells are involved in the response against extracellular bacteria, while Th2 cells are induced by helminths [61]. On the other hand, classes of differentiated T cells are immune-suppressive and act as a counter-regulatory system to maintain tolerance and immune system homeostasis. Two main classes of regulatory T cells have been characterized: regulatory T cells expressing the transcription factor FoxP3 (Tregs) and IL-10 secreting type 1 regulatory T cells (Tr1) [62,63]. Tregs originate either from the thymus immediately after birth (naturally induced Tregs, nTregs) [64] or by differentiating from naïve T cells in peripheral organs, especially in the gut (pTregs) in the presence of retinoic acid (RA) and TGF-beta [65,66]. nTregs have been involved in the maintenance of tolerance toward self-antigens, while pTregs are believed to maintain tolerance toward intestinal microbiota and antigens normally present in the gut lumen. Indeed, Tregs are able to control the intestinal antigen-driven inflammation induced by the adoptive transfer of naïve T cells in SCID mice [67]. Moreover, several lines of evidence indicate that microbiota products induce pTregs in the gut [68,69,70], and Tregs generated in vitro by TGF-beta are also able to control inflammation induced by the adoptive transfer of naïve T cells into RAG1-deficient mice [71], thus linking pTreg generation to the maintenance of immune homeostasis in the gut. Tr1 is another class of suppressive cells characterized by the expression of IL-10, by low levels of IFN-gamma, and by a variable amount of FoxP3 that is not required for their suppressive activity [72]. 

While the role of Tregs and Tr1 cells in the maintenance of immune system homeostasis and tolerance in the gut has been extensively investigated [73], their role in the resolution of inflammation is less well defined. The adoptive transfer of Tregs in colitic mice reduced intestinal inflammation [74]. In IBD patients, intravenous infusion of ovalbumin responsive Tregs induced a clinical response in 40% of treated patients, but the effect was transient. The limited efficacy shown in this trial might be explained by an insufficient gut-homing and anti-inflammatory capacity of the transferred cells. In an attempt to circumvent these issues, a new approach based on the isolation from peripheral blood and in vitro expansion of CD4+CD25+CD127lowCD45RA+ resting Tregs was developed [75]. These cells were stable in vitro, expressed alpha4/beta7 integrin and CCR6, and accumulated in the gut. However, data in IBD patients have not yet been reported.

In models of acute inflammation such as acute peritonitis, acute lung injury, and atherosclerosis, in which decreased Tregs and defective efferocytosis drive disease progression, the accumulation of Tregs at the site of inflammation was required to enhance efferocytosis in an IL-13-dependent manner. In these models, Treg-derived IL-13 induced IL-10 expression in macrophages, which in turn enhanced engulfment of apoptotic neutrophils and macrophage reprogramming, acting in an autocrine and paracrine manner [76]. Despite Treg accumulation during active IBD [77,78,79] and IL-13 [80] and IL-10 [81,82,83] upregulation in the gut of these patients, a clear demonstration that this pro-resolving mechanism operates in the gut mucosa is still missing.

**Figure 2:**
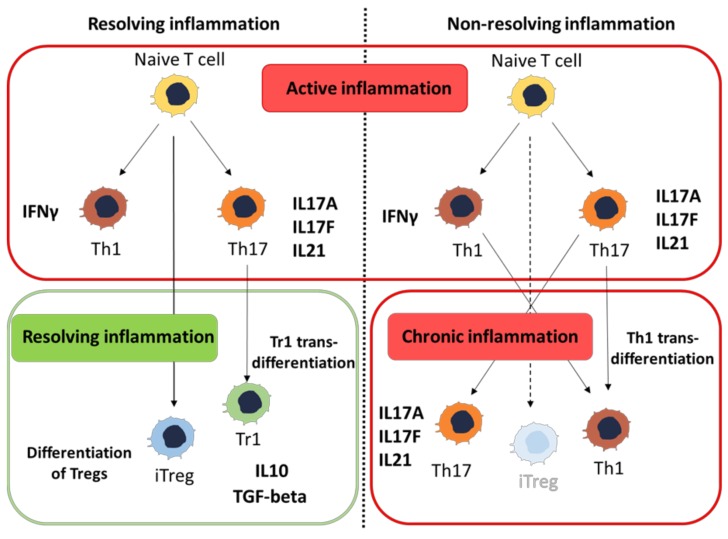
The role of peripheral induction of regulatory T cells (Tregs) and trans-differentiation of T helper 17 (Th17) cells in Tr1 or Th1 cells in inflammation resolution.

The trans-differentiation of highly pathogenic Th17 into anti-inflammatory Tr1 cells has also been associated with the resolution of intestinal inflammation. In a model of self-limiting colitis induced by the injection of anti-CD3 in mice, the resolution of the Th17-driven inflammation was associated with the trans-differentiation of Th17 cells into Tr1 cells, a phenomenon characterized by the downregulation of IL-17A and RORγt and the increased expression of IL-10. On the other hand, IL-10R-deficient mice treated with anti-CD3 failed to resolve intestinal inflammation, and Th17 failed to trans-differentiate into Tr1 cells and acquired a pro-inflammatory Th1 phenotype characterized by the expression of IFN-gamma [84]. These data indicate that T cell phenotype plasticity, and the capacity of a pro-inflammatory Th17 cells to acquire a regulatory phenotype, might be pivotal in the resolution of the inflammatory process in the gut (Figure 2).

Beside T cells, a subset of immune-suppressive and anti-inflammatory innate-lymphoid cells (iLC), called regulatory innate lymphoid cells (iLCregs), contributes to intestinal immune homeostasis and resolution of inflammation [85]. Indeed, the rapid expansion of pro-inflammatory iLC1 and iLC3 at the beginning of the inflammatory process is followed by an increase of iLCreg cells that suppress the expression of IFN-gamma and IL-17A but not of IL-22, which is important to maintaining intestinal barrier function. Ablation of iLCregs prolonged inflammation in mouse models of self-resolving colitis. The suppressive effect induced by iLCreg was dependent on the expression of IL-10 but not that of TGF-beta. In contrast, the autocrine and paracrine secretion of TGF-beta was required for the expansion and survival of iLCregs during the inflammatory response [85].

## 6. Concluding Remarks

In the last decades, many mechanisms involved in the inflammatory process have been characterized. Cell types belonging to innate and adaptive immunity, such as cytokines and chemokines, have been shown to play crucial roles in the initiation and perpetuation of the inflammatory process, and each element in this process has been evaluated as a potential target of anti-inflammatory and immunosuppressive therapies. In IBD, since the introduction of anti-TNF-alpha therapy in the late 1990s, several cytokines have been targeted by biologics and their efficacy tested in clinical trials [86]. More recently, new drugs targeting immune cell homing have been developed or are under development with promising results [87]. Despite the efficacy shown in randomized clinical trials and in clinical practice, all these new drugs show a loss of response over time and inflammation relapses when suspended. A possible explanation might be found in the pro-inflammatory nature of the target. Indeed, the blockage of just one of the pro-inflammatory mediators involved in the process might be circumvented by the activation of alternative, redundant pro-inflammatory pathways, thus leading to loss of response. Moreover, the transient blockage of pivotal intermediates in the inflammatory cascade does not necessarily restore all those systems (e.g., epithelial barrier, antigen tolerance) which have been damaged and/or altered during the inflammatory process and which are essential to maintaining the restored homeostasis, thus causing early relapses.

Few therapies have been tested in which the mechanism of action consisted of the restoration of a pro-resolving pathway. TGF-beta is a potent endogenous immunosuppressive cytokine acting on virtually all cells involved in the inflammatory process [88]. The immunosuppressive effect of TGF-beta, which is overexpressed in IBD, is blunted by the intracellular factor Smad7 overexpressed in these patients. Smad7 suppression by antisense oligonucleotide restored TGF-beta intracellular signaling in target cells, leading to resolution of the inflammatory process in animal models of colitis [89,90,91]. Initial data from phase I and II trials showed that the Smad7-antisense oligonucleotide Mongersen was able to induce long-lasting remission in patients affected by moderately active ileal Crohn’s disease after short-term therapy, suggesting that the re-activation of this pro-resolving pathway might lead to deeper suppression of the inflammatory process [92,93]. However, these results were not confirmed in a larger phase III trial, and the development of this drug was discontinued.

Engineered Tregs have been tested in human IBD with limited results [94], but the development of new strategies to expand autologous Tregs able to efficiently reach the intestinal site of inflammation remains a field of active research [75].

Finally, although clinical trials testing the efficacy of diet supplementation with o-3 PUFA (DHA or EPA) in IBD have provided discordant results [95], the effect of specific pro-resolving molecules (i.e., maresins, resolvins, protectins) derived from DHA and EPA metabolism has not yet been tested in IBD patients.

In recent years, many counter-regulatory mechanisms have started to be unveiled. Although many of these mechanisms have been investigated in animal models of colitis and they wait to be confirmed in human inflammatory bowel disease, they certainly represent a new potential therapeutic approach. Moreover, more mechanisms have yet to be discovered, and the future challenge will be to have an integrated view of the different axes involved in the resolution of inflammation.

Pro-inflammatory and pro-resolving mechanisms represent two opposing faces of the same process. Understanding the pathways leading to resolution of inflammation will provide a background for the development of effective strategies to treat chronic inflammatory diseases including IBD.

## Figures and Tables

**Table 1:** The role of SPMs in experimental models of colitis. Maresin (Mar); Protectin (PD), Resolvin (Rv), Dextran sulphate sodium (DSS), 2,4,6-Tri-nitro-benzene sulphonic acid (TNBS).

Reference	SPM	Model of Colitis	Outcome
Wang H et al. [96]	Mar1	IL10−/− mice	Decrease of colitis severity and pro-inflammatory cytokine expression.
Gobetti T et al. [27]	PD1 and RvD5	DSS in mice	Decrease of colitis severity and lymphocyte adhesion to the endothelium.
Bento AF et al. [97]	RvD1, RvD2	DSS and TNBS in mice	Decrease of disease index, pro-inflammatory cytokines, and adhesion molecules.
Ishida T et al. [98]	RvE1	DSS in mice	Decrease of colitis severity and pro-inflammatory cytokines
Arita M et al. [99]	RvE1	TNBS in mice	Improvement of histologic score, decrease of leukocyte infiltration and pro-inflammatory cytokine expression.
Marcon R et al. [25]	Mar1	DSS in mice	Decreased of inflammation, pro-inflammatory cytokine expression, and adhesion molecules.
